# Remineralization Potential of Three Restorative Glass Ionomer Cements: An In Vitro Study

**DOI:** 10.3390/jcm12062434

**Published:** 2023-03-22

**Authors:** James Ghilotti, Icíar Fernández, José Luis Sanz, María Melo, Carmen Llena

**Affiliations:** Department of Stomatology, Faculty of Medicine and Dentistry, Universitat de València, Gascó Oliag 1, 46010 Valencia, Spain

**Keywords:** resin-modified glass ionomer cements, remineralization, Fourier Transformed Infrared Spectroscopy, energy dispersive X-ray spectroscopy

## Abstract

The aim of this in vitro study was to evaluate the remineralizing ability of three glass ionomers on demineralized dentin with different thicknesses and time periods. Fifty third molars were obtained and were sectioned into 1-, 2-, and 3-mm thick slices (n = 36 for each thickness). The specimens were demineralized with 18% EDTA for 2 h. From the glass ionomer cements (GICs) under study (Ketac Molar Aplicap, Equia Forte, or Riva Light Cure), 1 mm was placed over each slice, set, and preserved in PBS until observation after 1, 7, 14, and 28 days after placement. For each material, thickness, and time, three samples were prepared. Using Fourier Transform Infrared Spectrometry (FTIR), apatite formation was determined on the side opposite to that on which the material had been placed. By means of Energy Dispersive Spectroscopy (EDX), the changes in the Calcium/Phosphate (Ca/P) ratio were evaluated. These changes were compared between the different materials by means of a two-way ANOVA test, considering time and dentin thickness, for a significance level of *p* < 0.05. Results: FTIR showed a peak at 1420 cm^−1^, evidencing the presence of carbonated hydroxyapatite in all the materials after 14 days, which indicates that a remineralization process occurred. Riva Light Cure showed the most homogeneous results at all depths at 28 days. The Ca/P ratio was maximum at 7 days in 2 mm of dentin for Riva Light Cure and Equia Forte HT (3.16 and 3.07; respectively) and for Ketac Molar at 14 days in 1 mm (3.67). All materials induced remineralization. Equia Forte achieved the greatest effect at 2 mm and Ketac Molar at 1 mm, whereas Riva Light Cure showed similar results at all depths. In terms of Ca/P ratio, Equia Forte and Riva Light Cure remineralized best at 2 mm, whereas for Ketac Molar, it was 1 mm. Carbonate apatite formation was higher at 24 h and 7 days for Ketac Molar, whereas it decreased at 14 days for Ketac Molar and peaked in Riva Light Cure and Equia Forte.

## 1. Introduction

Dentin is an organic, highly mineralized tissue that, together with the pulp, forms the dentin–pulp complex. It is made up of 70% minerals, with 20% organic content, and the remaining is 10% water [1]. It is formed by a network of collagen fibers on which hydroxyapatite crystals are deposited [2].

Caries is the most common cause of dentin destruction [3]. The existence of a correlation between histopathological findings and the clinical behavior of dentin in caries is elucidated in the research by Fusayama et al., who concluded that the color and hardness of carious dentin does not correspond to its degree of bacterial invasion [4]. 

The therapeutic approach to the carious lesion, from a minimally invasive perspective, has changed the traditional concept of “non-selective dentin removal”, until hard dentin is reached in the entire cavity, to the concept of “selective dentin removal”. The latter involves the removal of all or part of the soft dentin (depending on the depth of the cavity), leaving the leathery and firm dentin, and treating it appropriately to achieve remineralization [5]. Leathery and firm dentine correspond histologically to partially mineralized dentin, which has been classically called “affected dentin” [6]. In this area, the hydroxyapatite crystals are shorter, and the collagen fibers are not denatured, maintaining their characteristic bands [4]. 

Remineralization is defined as the net gain of calcified material in tooth structure, which replaces that previously lost through demineralization. The conventional strategy involves the use of solutions containing calcium and phosphate ions in the presence of various concentrations of fluoride. This occurs by the growth of hydroxyapatite crystals in partially demineralized dentin [7]. From a clinical point of view, it is of great importance to know the depth to which the GICs can act in order to be able to assess the thickness of demineralized dentin that can be maintained in the selective removal of caries.

Dentin tissue recovery is complex because it involves the reconstitution of two different phases: on the one hand, organic type I collagen, and on the other hand, inorganic apatite, both linked in a specific spatial relationship. This implies that remineralization alone is insufficient for the total recovery of demineralized dentin, so it is also necessary to restore the structure of the collagen matrix and for both phases to be linked in a specific manner [8].

Glass ionomer (or polyalkenoate) cements (GICs) belong to a group of materials based on an acid–base setting reaction, in which a weak acid reacts with a basic glass powder. They usually contain different proportions of polymeric acids such as polyoalkenoic acid, homopolymers (acrylic acid), and a 2:1 copolymer of polyacrylic acid and maleic acid. Regarding glass, it is essential that it has a basic nature. Its essential component is calcium-fluoro-alumino-silicate powder, but it can also contain phosphate, sodium, calcium, or strontium. Water is the third essential element. Its main function is to serve as a solvent for the polymeric acids, allowing them to act as a proton releaser. This is crucial for the setting reaction of GICs [9]. Some GICs contain additional chelators such as tartaric acid or citric acid to prevent the precipitation of aluminum salts by avoiding the premature formation of ionic cross-links [10].

Resin-modified glass ionomer cements (RMGICs) incorporate resin monomers which add a light curing reaction to the material. The proportion of resin in the material modifies its physical, mechanical, and biological characteristics and properties. RMGICs are described to be “dual-cured”, where resin monomers undergo photopolymerization upon light curing, and the GIC component is chemically cured in an acid–base reaction. The polymerized resin acts as a bridge and strengthens the material [11]. Biomineralization uses biomimetic analogs of dentin matrix proteins to induce the formation of amorphous calcium phosphate (ACP) nano-precursors in the internal compartments of collagen fibrils. This biomimetic remineralization process represents an approach based on creating nanocrystals that are small enough to fit into the gap zones between adjacent collagen molecules and establish a hierarchical order in the mineralized collagen [12].

To study the remineralization ability of dental materials, Fourier Transform Infrared Spectroscopy (FTIR), among other methods, can be used. This spectroscopic analysis method allows determining the nature of the mineral component of samples and provides quantitative information on the changes in mineral and matrix composition as mineralization takes place [13,14]. Energy Dispersive Spectroscopy (EDX), on the other hand, is used to analyze the chemical elements found on the surface of dentin once it has been treated with different remineralizing agents [15,16].

The rationale of the present work focuses on evaluating the remineralizing potential of different restorative GICs on different dentin thicknesses, since, in the literature, there is little information on both the remineralizing potential and the depth of remineralization achieved.

The aim of the present in vitro study is to evaluate the remineralization potential of three GICs at different depths of artificially demineralized dentin by analyzing their ability to form hydroxyapatite and by means of a chemical element analysis. The null hypothesis is that the remineralizing ability of the studied materials will not differ among them or at different times and dentin thicknesses. 

## 2. Materials and Methods

### 2.1. Sample Preparation

The study protocol was approved by the Research Ethics Committee of the University of Valencia (ID: 2030322). A total of 50 impacted wisdom teeth extracted surgically for medical reasons were selected. After extraction, the teeth were stored in 0.1% Thymol solution at 4 degrees Celsius for 24 h. Organic debris was removed, and they were preserved in Phosphate Buffer Solution (PBS) until their use in the study, no more than 3 months after extraction.

The teeth were embedded in epoxy resin (Resin Pro^®^, Barcelona, Spain) and kept at room temperature for 24 h until complete setting. Subsequently, the resin blocks were cut longitudinally with the Mintron Stuers^®^ precision cutting machine (Madrid, Spain) to obtain 1-, 2-, and 3-mm thickness sections with dentin on both sides of the cut, making a total of 114 cuts (n = 36 for each of the above-mentioned thicknesses, 6 to be used as controls (3 as positive controls and 3 as negative controls)). The samples for the positive controls were directly preserved in Phosphate Buffered Saline (PBS). The samples for the negative controls were demineralized, as was done with the study samples (described below), and then preserved in PBS.

After obtaining the sample sections, the study samples and the three samples used as negative control were demineralized. The demineralization process was as follows: the samples were immersed in 18% Ethylene Diamine Tetraacetic Acid (EDTA) solution (Clarben, Fuenlabrada, Madrid, Spain) for 2 h, after which, they were washed with distilled water and then dried. The GICs tested in the study, their compositions, and batch numbers are shown in Table 1.

**Figure 1:**
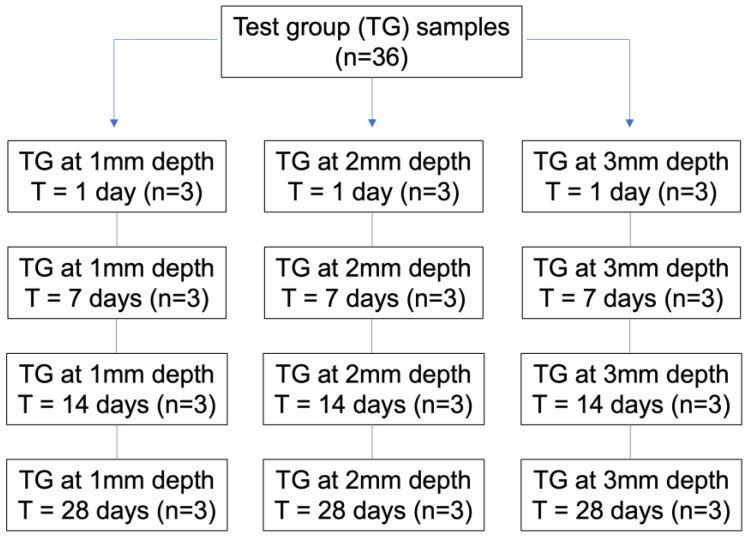
Flowchart representing the different test groups. Three test groups (TG) were assessed (KM, EF, and RL (n = 36 each)). Three different dentin depths were tested (1, 2, and 3 mm). Four different time points were tested (1, 7, 14, and 28 days).

**Figure 2:**
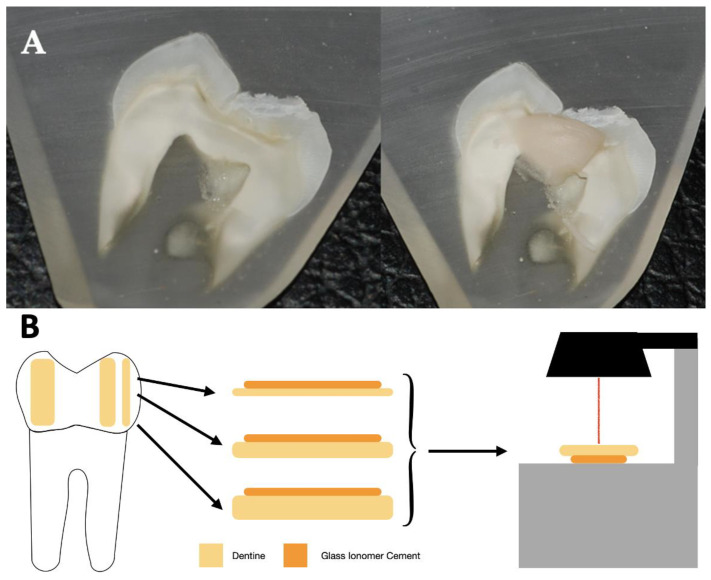
(**A**) Sample before and after the application of the GIC. (**B**) Schematic illustration of the sample preparation and analysis.

GICs were handled according to the manufacturer’s instructions. Ionomer (1 mm) was applied on each of the dentin samples, as shown in Figure 1. After applying the materials, EF and RL were light-cured for 40 seconds with an LED-B lamp (Guilin Woodpecker Medical Instrument, Guilin, Guangxi, China). KM was left 3.30 min to complete its polymerization. All samples were immersed in 2 mL of PBS for preservation until the time of measurement. Three specimens of each sample were prepared. Both the storage and characteristics of the samples were based on the methodology of previous studies [17,18,19]. The sample preparation is illustrated in Figure 2.

Parallelly, four 1-mm blocks were obtained for each pure material and time period, which were also preserved in PBS. 

Subsequently, and after each time period, the changes in the mineral composition of the dentin on the side opposite to that on which the material had been placed were analyzed. This was performed to assess the remineralization potential of the tested GICs at greater depths of dentin and not superficially. This is remarkable because, to our knowledge, no previous study has assessed the remineralization potential in depth.

### 2.2. Fourier Transform Infrared Spectrometry (FTIR)

This spectroscopic analysis technique is used to understand the structure of individual molecules and the composition of molecular mixtures. In the present study, it was used to determine the changes in the structure and composition of demineralized dentin that occurred on the side opposite to that on which the material was placed. This was done to evaluate the remineralization in depth induced by the different materials in demineralized dentin at different study times. The Cary 630 FTIR equipment (Agilent Technologies^®^ California, CA, USA) was used for this purpose. In this case, it was FTIR with attenuated reflectance (FTIR-ATR) by absorbance. 

The analysis of the samples with this technique produced graphs that provide information regarding the degree of light absorbance of the sample (in our case, dentin), corresponding to a particular type of compound. The intervals of interest for our study were [10]:

- 3000–3700 cm^−1^: Corresponding with OH group.
- 1636 cm^−1^: Corresponding with COO^−^ group.
- 1021 cm^−1^: Corresponding with PO_4_ groups.
- 820 and 1420 cm^−1^: corresponding with CO_3_ group.
- 700 cm^−1^: Corresponding with P_2_O_7_ group.
- The joint presence of PO_4_ and CO_3_ together indicates the presence of carbonate hydroxyapatite.

To analyze the composition of the pure material, the blocks of pure material were crushed to facilitate the measurement process. The spectra recorded the absorbance between 650 cm^−1^ and 4000 cm^−1^, and 64 scans were performed in each measurement with a resolution of 2 cm^−1^. Between each sample, 2-propanol was used to clean the surface and avoid contamination between samples.

### 2.3. Energy Dispersive Spectroscopy (EDX)

This is an analytical technique used for qualitative and quantitative elemental analysis. The aim of this technique was to determine the changes in the chemical composition of dentin, which was previously demineralized at a depth of 1, 2, and 3 mm, after contact with the different studied GICs. A Scanning Electron Microscope (SEM) (Hitachi S4800, Hitachi High-Technologies Corporation, Tokyo, Japan) with the XFlash 5030 system (Bruker, MA, USA) for EDX was used. Measurements were performed at 1000× magnification and a voltage of 10 kV.

### 2.4. Statistical Analysis

From the calcium (Ca) and phosphate (P) values obtained in the samples, the Ca/P ratio was calculated in the positive and negative control dentin samples and in the dentin treated with the different materials, thicknesses, and times. The percentage of remineralization was determined according to the increase in the Ca/P ratio in the treated dentin relative to the positive and negative controls The Kruskal Wallis test with the Dunn–Bonferroni post hoc method was applied, considering time and dentin thickness for each material. The significance level considered was *p* < 0.05. SPSS 28.0 statistical package (IBM, Chicago, IL, USA) was used.

## 3. Results

### 3.1. Fourier Transform Infrared Spectrometry (FTIR)

**Figure 3:**
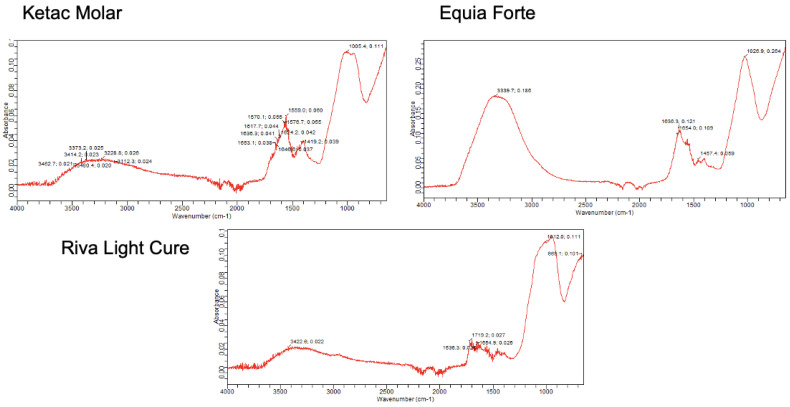
FTIR spectrum of the different pure materials 24 h after setting.

Figure 3 shows the plots corresponding to the pure material at 24 h. In Ketac Molar, the presence of an initial band around 3462 cm^−1^ stands out. Additionally, peaks were found near 1636 cm^−1^, corresponding to COO^−^, followed by a peak at 1419 cm^−1^, corresponding to the presence of CO_3_. Lastly, a peak at 1005 cm^−1^, corresponding to the presence of PO_4_ groups, was observed, which indicates the presence of carbonated hydroxyapatite in the material. 

For Equia Forte, a marked peak was observed at 3339 cm^−1^, corresponding to the presence of OH groups, and a peak at 1636 cm^−1^, corresponding to COO^−^, followed by a peak at 1457 cm^−1^ and a peak at 1026 cm^−1^. Together, they indicate the presence of carbonate hydroxyapatite.

In the case of Riva Light Cure, an initial band was found at 3422 cm^−1^, indicative of the presence of OH groups. A peak was also found at 1636 cm^−1^ (corresponding to the presence of COO^−^), followed by another peak at 1020 cm^−1^, which represents the presence of PO_4_ groups. Finally, a peak was observed at 667 cm^−1^, indicating the presence of P_2_O_7_. 

**Figure 4:**
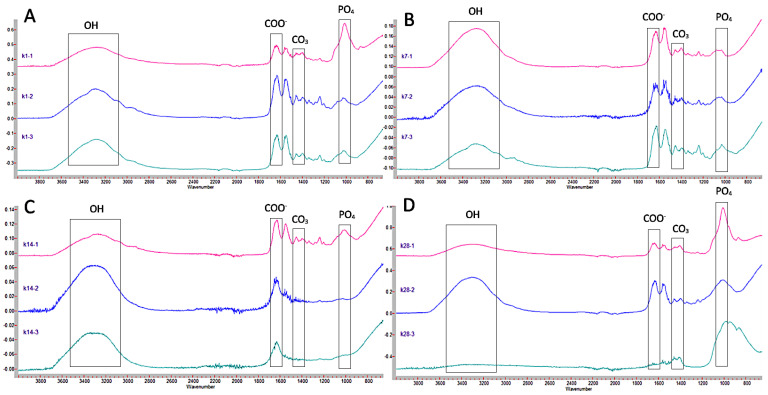
Comparison of Ketac Molar FTIR spectra at different thicknesses (1, 2, and 3 mm) at 24 h (**A**), 7 days (**B**), 14 days (**C**), and 28 days (**D**). The peaks of the OH, COO^−^, CO_3_, and PO_4_ groups of interest in this study are indicated.

Figure 4 shows the graphs corresponding to Ketac Molar for the different thicknesses and times. It can be seen how at 24 h (Figure 4A), the absorbance values maintained a similar pattern in the three dentin thicknesses, highlighting only that the peak corresponding to carbonated hydroxyapatite (1399 cm^−1^) is more pronounced in the 1 mm thick sample compared to the other two. At 7 days, the patterns were similar in the three thicknesses (Figure 4B). At 14 days (Figure 4C), it is noteworthy that at depths of 2 and 3 mm, the level of water absorption was lower (1636 cm^−1^), and the presence of the other elements decreased compared to the 1 mm thick sample. At 28 days (Figure 4D), the graphs show a similar arrangement. 

**Figure 5:**
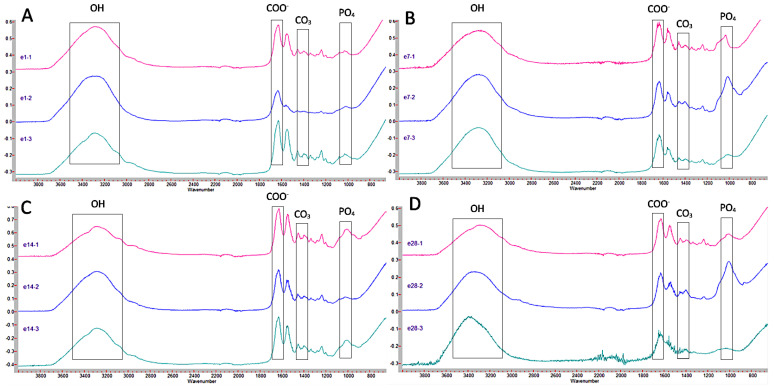
Comparison of Equia Forte FTIR spectra at different thicknesses (1, 2, and 3 mm) at 24 h (**A**), 7 days (**B**), 14 days (**C**), and 28 days (**D**). The peaks of the OH, COO^−^, CO_3_, and PO_4_ groups of interest in this study are indicated.

Figure 5 shows the graphs regarding Equia Forte. At 24 h (Figure 5A), in all thicknesses, the behavior is similar: a peak corresponding to the presence of OH bonds, followed by a peak corresponding to water absorption, then several peaks close to 1420 cm^−1^, corresponding to CO_3_, as well as a peak at 1031 cm^−1^, close to 1021 cm^−1^, corresponding to the presence of PO_4_ groups. At 7 and 14 days (Figure 5B,C), the graphs are also similar, with peaks similar to those found at 24 h. At 28 days (Figure 5D), at 2 mm, the peak indicative of the presence of PO_4_ is more pronounced compared to the other two graphs, although at 1 mm thickness, it is still a notable peak. However, at 3 mm, it is practically not marked. 

**Figure 6:**
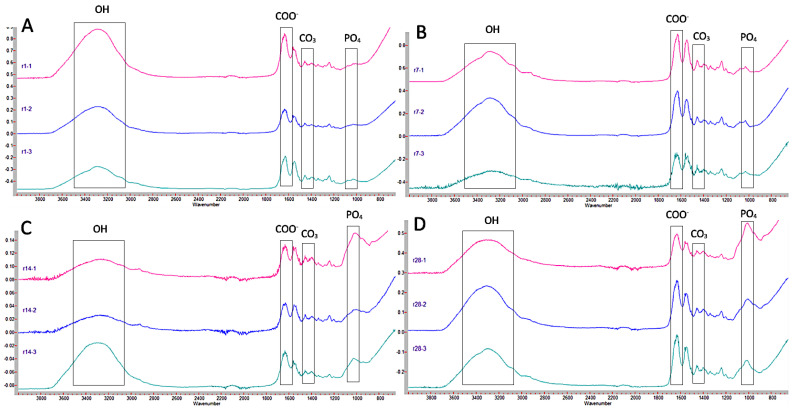
Comparison of Riva Light Cure FTIR spectra at different thicknesses (1, 2, and 3 mm) at 24 h (**A**), 7 days (**B**), 14 days (**C**), and 28 days (**D**). The peaks of the OH, COO^−^, CO_3_, and PO_4_ groups of interest in this study are indicated.

Figure 6 shows the graphs regarding Riva Light Cure. At 24 h (Figure 6A), there is a peak at 3287 cm^−1^, corresponding to the presence of OH groups, followed by a peak at 1628 cm^−1^, corresponding to COO^−^ groups. Several peaks with values at 1457 cm^−1^, 1399 cm^−1^, or 1340 cm^−1^ can also be seen, associated with the presence of CO_3_. Finally, a peak can be seen at 1031 cm^−1^, corresponding to the presence of PO_4_ groups. At 7 days (Figure 6B), an initial section corresponding to COO^−^ (1636 and 1599 cm^−1^) is observed, followed by a peak at 1340 cm^−1^ and 1457 cm^−1^, indicative of the presence of CO_3_, and a peak at 1033 cm^−1^, corresponding to the presence of PO_4_ groups. At 14 and 28 days, the results are similar (Figure 6C,D).

**Figure 7:**
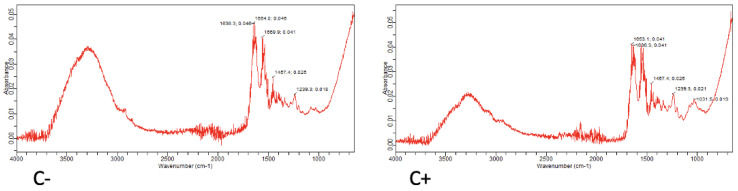
FTIR spectrum of the negative control (C−), corresponding to demineralized dentin, and positive control (C+), corresponding to healthy dentin.

The spectra of the positive (C+) and negative (C−) controls are shown in Figure 7. When analyzing the negative control (Figure 7, C− panel), an important initial peak is found, corresponding to the presence of OH groups, followed by a peak at 1636 cm^−1^, which indicates the presence of COO^−^ groups, and a peak at 1457 cm^−1^, corresponding to the presence of CO_3_. In the graph corresponding to the positive control (Figure 7, C+ panel), the disposition is similar. The initial peak, which corresponds to the presence of OH, is less marked, but the peak at 1636 cm^−1^ appears again, corresponding to the presence of COO^−^. The peaks at 1457 and 1239 cm^−1^ also appear, indicative of the presence of CO_3_. Finally, a peak at 1031 cm^−1^ can also be seen, which corresponds to the presence of PO_4_ groups.

### 3.2. Energy Dispersive Spectroscopy (EDX)

The Ca/P ratios (mol) were established for the control samples (1.47 for healthy dentin (positive control) and 1.40 for demineralized dentin (negative control)) and for the test groups (Table 2).

**Figure 8:**
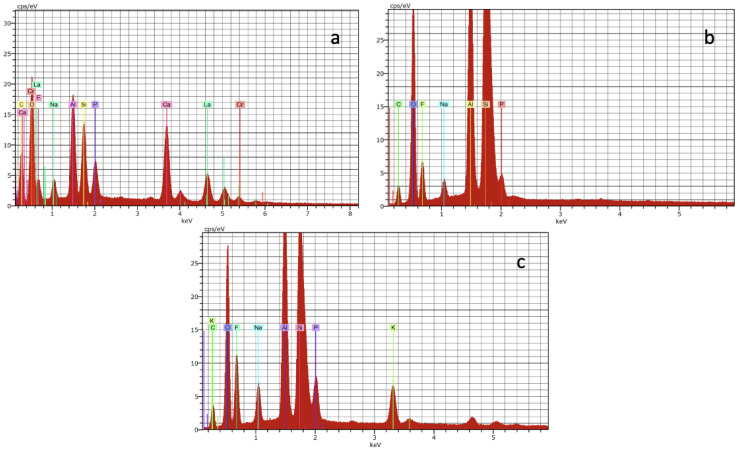
EDX analysis graphs of Ketac Molar (**a**), Riva Light Cure (**b**), and Equia Forte (**c**) pure at 28 days.

Figure 8 shows the graphs obtained from the EDX analysis of the pure materials at 28 days.

The percentage change in the Ca/P ratio (mol) was calculated for each of the materials by thickness and time compared to the positive and negative controls, respectively (healthy dentin, C+; and demineralized dentin, C−).

Table 3 shows the percentage change in the Ca/P ratio (mol) of dentin with the different materials, times, and dentin thicknesses compared to the non-demineralized dentin. In the dentin treated with Riva Light Cure and Equia Forte, the highest percentage of remineralization was found at 2 mm and 7 days after application, with no significant differences between them, nor with Ketac Molar at 1 mm at 7 days (*p* > 0.05). However, in Ketac Molar, the highest remineralization was found at 1 mm thickness at 14 days after application, significantly higher than that found in the other two materials (*p* < 0.05). At 3 mm, Riva Light Cure and Equia Forte did not show significant differences in Ca/P gain at 1, 7, and 14 days (*p* > 0.05). At 28 days, at 3mm depth, no gain was found with these materials. In Ketac Molar, Ca/P gain was always found at 1 mm, whereas at the remaining depths, the behavior was more erratic.

Table 4 shows the percentage of gain in the Ca/P ratio of dentin by the materials, times, and dentin thicknesses compared to demineralized dentin. It was also observed that in the dentin treated with Riva Light Cure and Equia Forte, the highest percentage of remineralization was found at 2 mm and 7 days after application, without significant differences (*p* > 0.05). However, in Ketac Molar, the highest remineralization was found at 1 mm 14 days after application, with significant differences compared to the other two materials (*p* < 0.05). Ketac Molar and Equia Forte did not show significant differences between the percentage increase in the Ca/P ratio at 1 mm between 1 day and 14 days after application (*p* > 0.05). The same occurred with Equia Forte at 2 mm and Riva Light Cure at 3 mm.

## 4. Discussion

Following the criteria of “minimal intervention” are the techniques of “selective caries removal” [20]. The application of these techniques requires the use of bioactive materials for the restoration. A bioactive material must induce a favorable response, which requires an interaction between the material and the dental tissues, leading to the formation of mineralized tissue [21]. Thus, the treatment of deep caries lesions involves the use of ion-releasing agents to induce the remineralization of demineralized dentin through the formation of apatite [22].

FTIR is a spectroscopic assay by which the remineralizing potential of a material can be examined, as it allows the identification of different functional groups. The presence of peaks in the zone around 1020 cm^−1^ indicates the presence of phosphate groups, which, in this case, are usually PO_4_ groups. The presence of other peaks in the 1420 cm^−1^ region indicates that carbonate groups such as CO^3^ are present. The association of these two peaks implies the presence of carbonated hydroxyapatite, which is expected in the dentin remineralization process [23,24,25,26,27].

After the analysis of the results, the null hypothesis was rejected, since materials, time, and dentin thickness influenced the remineralizing ability of the different GICs included in the present study. 

After 1 day, only Ketac Molar was able to form apatite and only to a depth of 1 mm. After 7 days, Ketac Molar achieved a homogeneous effect in all thicknesses, whereas Equia Forte only achieved remineralization up to 2 mm, with a slightly greater effect at 2 mm compared to 1 mm. Finally, Riva Light Cure showed slight peaks, but did not clarify the presence of apatite at any thickness. These results were expected, since Ketac Molar had already performed better in shorter periods.

After 14 days, all the materials showed a notable presence of carbonate apatite at 1 mm higher than the greater thicknesses, except in the case of Equia Forte, where a peak of greater intensity appears at 2 mm. It should be noted that in Ketac Molar, at 2 and 3 mm thicknessess, the presence of apatite is lower than at 7 days.

Finally, at 28 days, apatite was present at all depths with all materials. Equia Forte achieved a greater presence at 2 mm, whereas Ketac Molar did so at 1 mm. Riva Light Cure showed similar results at all depths. 

The non-linear remineralization behavior of Ketac Molar and Equia Forte should be highlighted. In Ketac Molar, the only constant is that its main effect is seen at 1 mm depth, whereas Equia Forte, at 7 and 28 days, shows more apatite formation at 2 mm. By contrast, Riva Light Cure shows an increase in apatite directly related to the exposure time, which was constant in the three dentin thicknesses. 

On the other hand, the SEM-EDX system allows knowing the amount of calcium and phosphate present in the sample, either in simple (the pure element) or complex form (as part of a more complex compound). With this information, the Ca/P ratio of each of the analyzed samples can be established.

According to other published studies, the increase in the Ca/P ratio is an important indicator of remineralization [28], since it allows establishing whether a material can be effective when applied on demineralized dentin in accordance with current trends of minimal intervention. 

Regarding the studied materials, it is noteworthy that Ketac Molar performed better at 1 mm thickness, whereas Riva Light Cure and Equia Forte showed better results at 2 mm. This indicates that the latter two materials can remineralize at a greater depth than the former. However, at 3 mm thickness, all the materials show a decrease in the Ca/P ratio, highlighting that at 28 days, both Equia Forte and Riva Light Cure show a Ca/P ratio below that of the control. As such, at this depth and time, no remineralization is showed by the samples. 

The fact that one of the studied GICs incorporates resin in its composition (Riva Light Cure) did not affect dentin remineralization, neither in terms of hydroxyapatite formation nor the Ca/P ratio found at different depths. Its behavior was very similar to that obtained by Equia Forte, which does not incorporate resin. Resin incorporation did not reduce the remineralization potential and depth achieved. Thus, from a clinical point of view, the use of a RMGIC that in addition to the chemical bonding (achieved by polycarboxylates), can also achieve micromechanical interlocking in hybridized dentine through the infiltration of the collagen network that is exposed by polyacrylic acid [29], is a good alternative in minimally invasive dentistry procedures.

When focusing on other elements, a striking fact is that despite being glass ionomers, which are materials that contain fluoride [30], in our samples, it was only detected in pure material samples, as shown in Figure 8. Its presence in the samples at 1, 2, and 3 mm thickness was detected sporadically at certain times and thicknesses and did not follow any common pattern. Consequently, this could be a subject for future study.

Strontium is indicated as present in the chemical formulation of Riva Light Cure and Equia Forte by their respective manufacturers. However, it was not detected in Equia Forte in any case. In Riva Light Cure, it was only detected at 28 days in 1 mm thickness and in the pure material. Strontium has chemical and physical properties similar to those of calcium, so it is theoretically capable of replacing Ca in hydroxyapatite [31]. Studies using strontium-doped bioactive glasses show less demineralization of enamel and dentin, but no better remineralization than bioactive glasses without strontium [32].

Regarding the limitations of the present study, a series of points should be highlighted: firstly, its inherent in vitro nature, which limits the application of the results to the clinical setting and requires further evidence to confirm the reported results. Secondly, the absence of a standardized methodology for this type of studies results in a substantial heterogeneity among similar studies. For example, the sample size for test groups varies among studies in the field [16,18,19,33]. In the present study, a sample size of n = 3 was selected for each group. The low sample size, both in the present study and similar studies, may reduce the representativeness of the results. Thus, results should be interpreted as a preliminary assessment of the remineralization ability of GICs at a laboratory level and should serve as a base for future studies.

Nevertheless, the results of this study can guide clinicians in the thickness of demineralized dentin that can be left in the cavity, as well as in the choice of GIC, depending on the clinical situation. On occasions where a rapid remineralization effect is required in minimal thicknesses, Ketac Molar will be suitable, whereas if the objective is to achieve a deeper remineralization, Riva Light Cure and Equia Forte show better results. 

## 5. Conclusions

Carbonate apatite was found by FTIR at 28 days at all depths with all tested materials. Equia Forte achieved the greatest effect at 2mm depth, whereas Ketac Molar achieved the greatest effect at 1 mm. Riva Light Cure showed similar results at all depths. 

In terms of Ca/P ratio, Equia Forte and Riva Light Cure showed similar results at all depths. Equia Forte and Riva Light Cure showed a higher dentin remineralization at 2 mm thickness, whereas Ketac Molar performed better at 1 mm thickness. 

In terms of time, the formation of carbonate apatite was higher at 24 h and 7 days for Ketac Molar. However, at 14 days, it was reduced in this material and reached its peak in Riva Light Cure and Equia Forte.

According to the results of this study, long-term remineralization was not constant at 3 mm, making it inadvisable to leave such a large thickness of demineralized dentin.

## Figures and Tables

**Table 1:** Composition, manufacturer, and batch number of the studied GICs.

Material	Composition	Manufacturer	Batch N°
Ketac Molar Aplicap (KM)	Liquid: water, copolymer, polyacrylic acid, maleic and tartaric acid Powder: aluminum-calcium-lanthanum fluorosilicate glass	3M ESPE^®^ Seefeld, Germany	56420
Equia Forte HT (EF)	Liquid: polycarboxylic acid, water Powder: strontium fluoro aluminum silicate glass, iron oxide	GC Corp^®^, Tokio, Japan	901584
Riva Light Cure HV (RL)	Liquid: polyacrylic acid, tartaric acid, HEMA Powder: bioactive glass (Ionglass™), contains fluoride and strontium ions.	SDI^®^ Victoria, Australia	873003

**Table 2:** Ca/P ratio (mol) calculated from EDX results by material, thickness, and time.

	1 Day	7 Days	14 Days	28 Days
KM 1 mm	2.23 ± 0.6	2.46 ± 0.7	2.84 ± 0.9	1.85 ± 0.8
KM 2 mm	1.52 ± 0.3	1.24 ± 0.2	1.93 ± 0.4	1.76 ± 0.5
KM 3 mm	1.17 ± 0.1	1.94 ± 0.4	2.12 ± 0.5	1.65 ± 0.6
EF 1 mm	1.09 ± 0.2	2.01 ± 0.7	1.01 ± 0.1	1.74 ± 0.5
EF 2 mm	2.21 ± 0.9	2.37 ± 0.8	2.12 ± 0.3	1.94 ± 0.2
EF 3 mm	1.91 ± 0.7	2.16 ± 0.6	1.72 ± 0.2	1.11 ± 0.4
RL 1 mm	1.98 ± 0.6	1.34 ± 0.5	1.83 ± 0.4	2.01 ± 0.5
RL 2 mm	1.65 ± 0.5	2.44 ± 0.7	1.69 ± 0.5	1.94 ± 0.6
RL 3 mm	2.09 ± 0.6	1.94 ± 0.3	1.72 ± 0.3	1.11 ± 0.2

**Table 3:** Percentage gain in Ca/P ratio (mol), calculated from EDX results, in dentin as a function of different materials, times, and thickness compared to non-demineralized dentin.

	1 Day	7 Days	14 Days	28 Days
KM 1 mm	34.10	40.15	48.18	20.54 ^a^
KM 2 mm	3.50	−18.33	23.88	16.70 ^a^
KM 3 mm	−25.16	24.09	30.65	10.69
EF 1 mm	−35.05	26.89	−45.48	15.58
EF 2 mm	33.52	38.00	30.65	24.41
EF 3 mm	22.89	31.90	14.59	−32.68
RL 1 mm	25.63 ^a^	−9.59	19.49	26.91 ^a^
RL 2 mm	10.94	39.82	12.90 ^a^	24.41 ^a^
RL 3 mm	29.80 ^a^	24.09	14.59 ^a^	−32.68

The same letter in the superscript indicates no significant differences between thicknesses for each material and time.

**Table 4:** Percentage gain in Ca/P ratio (mol), calculated from EDX results, in dentin by materials, times, and thickness compared to demineralized dentin.

	1 Day	7 Days	14 Days	28 Days
KM 1 mm	37.24	43.00	50.64	24.33 ^a^
KM 2 mm	8.10	−12.69	27.51	20.66 ^a^
KM 3 mm	−19.20	27.70	33.96	14.94
EF 1 mm	−28.62	30.37 ^a^	−38.55	19.60
EF 2 mm	36.68	40.96	33.95	28.01
EF 3 mm	26.56	35.14 ^a^	18.66	−26.36
RL 1 mm	29.18	−4.37	23.32	30.40 ^a^
RL 2 mm	15.18	42.68	17.05 ^a^	28.01 ^a^
RL 3 mm	33.14	27.70	18.66 ^a^	−26.36

The same letter in the superscript indicates no significant differences between thicknesses for each material and time.

## Data Availability

The data presented in this study are available on request from the corresponding author.

## References

[1] Bertassoni L.E., Habelitz S., Kinney J.H., Marshall S.J., Marshall G.W.J. (2009). Biomechanical Perspective on the Remineralization of Dentin. Caries Res..

[2] Abou Neel E.A., Aljabo A., Strange A., Ibrahim S., Coathup M., Young A.M., Bozec L., Mudera V. (2016). Demineralization-Remineralization Dynamics in Teeth and Bone. Int. J. Nanomed..

[3] James S.L., Abate D., Abate K.H., Abay S.M., Abbafati C., Abbasi N., Abbastabar H., Abd-Allah F., Abdela J., Abdelalim A. (2018). Global, Regional, and National Incidence, Prevalence, and Years Lived with Disability for 354 Diseases and Injuries for 195 Countries and Territories, 1990–2017: A Systematic Analysis for the Global Burden of Disease Study 2017. Lancet.

[4] Fusayama T., Okuse K., Hosoda H. (1966). Relationship between Hardness, Discoloration, and Microbial Invasion in Carious Dentin. J. Dent. Res..

[5] Barros M.M.A.F., de Queiroz Rodrigues M.I., Muniz F.W.M.G., Rodrigues L.K.A. (2020). Selective, Stepwise, or Nonselective Removal of Carious Tissue: Which Technique Offers Lower Risk for the Treatment of Dental Caries in Permanent Teeth? A Systematic Review and Meta-Analysis. Clin. Oral. Investig..

[6] Innes N.P.T., Frencken J.E., Bjørndal L., Maltz M., Manton D.J., Ricketts D., van Landuyt K., Banerjee A., Campus G., Doméjean S. (2016). Managing Carious Lesions: Consensus Recommendations on Terminology. Adv. Dent. Res..

[7] Dai L., Liu Y., Salameh Z., Khan S., Mao J., Pashley D.H., Tay F.R. (2011). Can Caries-Affected Dentin Be Completely Remineralized by Guided Tissue Remineralization?. Dent. Hypotheses.

[8] Gao W., Smales R.J., Yip H.K. (2000). Demineralisation and Remineralisation of Dentine Caries, and the Role of Glass-Ionomer Cements. Int. Dent. J..

[9] Sidhu S.K., Nicholson J.W. (2016). A Review of Glass-Ionomer Cements for Clinical Dentistry. J. Funct. Biomater..

[10] Nicholson J.W., Brookman P.J., Lacy O.M., Wilson A.D. (1988). Fourier Transform Infrared Spectroscopic Study of the Role of Tartaric Acid in Glass-Ionomer Dental Cements. J. Dent. Res..

[11] Berzins D.W., Abey S., Costache M.C., Wilkie C.A., Roberts H.W. (2010). Resin-Modified Glass-Ionomer Setting Reaction Competition. J. Dent. Res..

[12] He L., Hao Y., Zhen L., Liu H., Shao M., Xu X., Liang K., Gao Y., Yuan H., Li J. (2019). Biomineralization of Dentin. J. Struct. Biol..

[13] Kong W., Du Q., Qu Y., Shao C., Chen C., Sun J., Mao C., Tang R., Gu X. (2022). Tannic Acid Induces Dentin Biomineralization by Crosslinking and Surface Modification. RSC Adv..

[14] Jang J.-H., Lee M.G., Ferracane J.L., Davis H., Bae H.E., Choi D., Kim D.-S. (2018). Effect of Bioactive Glass-Containing Resin Composite on Dentin Remineralization. J. Dent..

[15] Kranz S., Heyder M., Mueller S., Guellmar A., Krafft C., Nietzsche S., Tschirpke C., Herold V., Sigusch B., Reise M. (2022). Remineralization of Artificially Demineralized Human Enamel and Dentin Samples by Zinc-Carbonate Hydroxyapatite Nanocrystals. Materials.

[16] Sobh E.G., Hamama H.H., Palamara J., Mahmoud S.H., Burrow M.F. (2022). Effect of CPP-ACP Modified-GIC on Prevention of Demineralization in Comparison to Other Fluoride-Containing Restorative Materials. Aust. Dent. J..

[17] Gandolfi M.G., Taddei P., Siboni F., Modena E., De Stefano E.D., Prati C. (2011). Biomimetic remineralization of human dentin using promising innovative calcium-silicate hybrid “smart” materials. Dent. Mater..

[18] Sajini S.I., Alshawi B.A., Alharbi L.M. (2022). Assessment of remineralisation potentials of bioactive dental composite using an in-vitro demineralised dentine model. J. Taibah Univ. Sci..

[19] Liang K., Xiao S., Shi W., Li J., Yang X., Gao Y., Gou Y., Hao L., He L., Cheng L. (2015). 8DSS-promoted remineralization of demineralized dentin in vitro. J. Mater. Chem. B.

[20] Schwendicke F. (2017). Contemporary Concepts in Carious Tissue Removal: A Review. J. Esthet. Restor. Dent..

[21] de Oliveira Roma F.R.V., de Oliveira T.J.L., Bauer J., Firoozmand L.M. (2023). Resin-Modified Glass Ionomer Enriched with BIOGLASS: Ion-Release, Bioactivity and Antibacterial Effect. J. Biomed. Mater. Res. B. Appl. Biomater..

[22] Valizadeh S., Kamangar S.S.H., Nekoofar M.H., Behroozibakhsh M., Shahidi Z. (2022). Comparison of Dentin Caries Remineralization with Four Bioactive Cements. Eur. J. Prosthodont. Restor. Dent..

[23] Daneshpoor N., Pishevar L. (2020). Comparative Evaluation of Bioactive Cements on Biomimetic Remineralization of Dentin. J. Clin. Exp. Dent..

[24] Simila H.O., Karpukhina N., Hill R.G. (2018). Bioactivity and Fluoride Release of Strontium and Fluoride Modified Biodentine. Dent. Mater..

[25] Correa D., Almirall A., García-Carrodeguas R., dos Santos L., de Aza A.H., Parra J., Ángel Delgado J. (2014). β-Dicalcium Silicate-Based Cement: Synthesis, Characterization and in Vitro Bioactivity and Biocompatibility Studies. J. Biomed. Mater. Res. A.

[26] Chen B., Haapasalo M., Mobuchon C., Li X., Ma J., Shen Y. (2020). Cytotoxicity and the Effect of Temperature on Physical Properties and Chemical Composition of a New Calcium Silicate-Based Root Canal Sealer. J. Endod..

[27] Camilleri J. (2015). Sealers and Warm Gutta-Percha Obturation Techniques. J. Endod..

[28] Zhou Z., Ge X., Bian M., Xu T., Li N., Lu J., Yu J. (2020). Remineralization of Dentin Slices Using Casein Phosphopeptide-Amorphous Calcium Phosphate Combined with Sodium Tripolyphosphate. Biomed. Eng. Online.

[29] Song F.V., Yang B., Di Tommaso D., Donnan R.S., Chass G.A., Yada R.Y., Farrare D.H., Tian K.V. (2022). Resolving nanoscopic structuring and interfacial THz dynamics in setting cements. Mater. Adv..

[30] Kun V.T., Chass G.A., Di Tommaso D. (2016). Simulations reveal the role of composition into the atomic-level flexibility of bioactive glass cements. Phys. Chem. Chem. Phys..

[31] Thuy T.T., Nakagaki H., Kato K., Hung P.A., Inukai J., Tsuboi S., Nakagaki H., Hirose M.N., Igarashi S., Robinson C. (2008). Effect of Strontium in Combination with Fluoride on Enamel Remineralization in Vitro. Arch. Oral. Biol..

[32] Dai L.L., Mei M.L., Chu C.H., Lo E.C.M. (2021). Remineralizing Effect of a New Strontium-Doped Bioactive Glass and Fluoride on Demineralized Enamel and Dentine. J. Dent..

[33] Zhou Y.Z., Cao Y., Liu W., Chu C.H., Li Q.L. (2012). Polydopamine-induced tooth remineralization. ACS Appl. Mater. Interfaces.

